# Supplementary material for: Extracorporeal Magnetotransduction Therapy as a New Form of Electromagnetic Wave Therapy: From Gene Upregulation to Accelerated Matrix Mineralization in Bone Healing
Source: Biomedicines. 2024 Oct 7;12(10):2269. doi: 10.3390/biomedicines12102269 (PMC11505246; doi:10.3390/biomedicines12102269)
Supplement: Supplementary file 1 [file biomedicines-12-02269-s001.zip › biomedicines-3218478-supplementary.pdf]

Table S1: PCR primers used for our study. TaqManTM Assays name and Assay ID is listed.

TaqManTM Assays Thermo Fisher Scientific	
GAPDH	4333764F
RUNX2	Hs01047973ml
SP7	Hs01866874_m1
ALPL	Hs01029144_m1
COL1A1	Hs00164004_m1
BGLAP	Hs01587814_m1
SPP1	Hs00959010_m1
MEPE	Hs00220237_ml
PHEX	Hs01011689_m1
ENPP1	Hs01054040_m1
ENPP2	Hs00905117_m1
ENPP3	Hs013038393_m1
PHOSPHO1	Hs01370290_m1
